# Perceived Harm to Pet Health Associated with Human Quality of Life After a Public Health Disaster

**DOI:** 10.3390/ijerph22020250

**Published:** 2025-02-11

**Authors:** Diana K. Haggerty, Robert Wahl, Nicole Jones, Jenny LaChance, Mona Hanna

**Affiliations:** 1 Michigan State University–Hurley Children’s Hospital Pediatric Public Health Initiative, Charles Stewart Mott Department of Public Health, College of Human Medicine, Michigan State University, Flint, MI 48502, USA; 2 Department of Pediatrics and Human Development, College of Human Medicine, Michigan State University, Flint, MI 48502, USA

**Keywords:** Flint water crisis, quality of life, pet health, environmental health, public health emergency, One Health

## Abstract

This study’s goal was to evaluate associations of human exposure to unfiltered tap water during the Flint water crisis (FWC) with perceived harm to pets from exposure to contaminated water. We also explored the associations of perceived pet harm with participants’ self-reported general, physical, and mental health, as well as quality of life. Adult (*n* = 3264) pet owners from a public health registry reported unfiltered tap water exposure, perceived pet health, and general health/quality of life at baseline, as well as health/quality of life 1 year later (*n* = 1172). Using frequencies, percentages, and odds ratios, we evaluated associations of unfiltered tap water consumption with perceived pet health (cross-sectional) and perceived pet health with general health and quality of life (cross-sectional and longitudinal). Daily unfiltered tap water drinkers were 3.12 (95% CI: 2.33–4.23) times more likely to report the FWC had made their pet ill compared to participants who never drank unfiltered water. Participants who reported Flint water made their pet ill had approximately a two-fold increase in odds of reporting poor/fair across all four health/quality-of-life measures compared to those who did not. Both animals and humans were exposed and impacted by the FWC. This study supports the interconnectedness between human and animal health, especially regarding environmental disaster exposure and outcomes.

## 1. Introduction

One Health emphasizes the interconnectedness of the health of humans, animals, and the environment [1]. The approach has mainly been applied to the management of antimicrobial resistance and zoonoses [2]. However, the influence of pets on human health has been hypothesized to arise through multiple pathways [3,4], such as promoting physical activity and providing companionship [4,5]. Pets are important members of families, and the welfare of pets is an important determinant in decision-making and help-seeking during natural disaster and crisis situations [6,7] and the occurrence of post-disaster distress [5]. Pets may also serve as an early warning for environmental hazards that impact human health, such as exposure to contaminants at superfund sites [8], as pets may have larger exposures than their owners to contaminants in soil or house dust due to their proximity to the ground [9]. In addition, physiological responses to environmentally related diseases in pets are similar to human responses, and because of pets’ shorter lives, it may be easier to observe latency periods for some diseases. Ostrowski showed that dogs can act as sentinels of child blood lead levels when both reside in lead-contaminated environments [10]. A One Health approach can help us identify pet exposures and pet health during man-made, prolonged disasters, like the Flint water crisis (FWC), and better identify how they are associated with the long-term health outcomes of their owners.

The FWC was the result of a decision to switch the municipal water supply for the city of Flint, Michigan, from pre-treated Lake Huron water supplied by Detroit, Michigan, to the Flint River [11]. The failure of officials to implement appropriate corrosion control resulted in the exposure of more than 140,000 people to lead and other environmental contaminants for 18 months [12,13]. Along with humans, pets were also unknowingly exposed [14]. A study that evaluated blood lead concentrations of dogs exposed to the FWC found mean blood lead concentrations were three parts per billion higher in Flint dogs compared to the control population (median Flint dogs: four parts per billion; median control dogs: one part per billion) and 8.5% of the FWC-exposed dogs had blood lead concentrations of 20 parts per billion or higher (control dogs: 2.1%) [15]. Due to the potential for lead exposure to cause chronic health conditions [16], and the connections between pet and human health, we hypothesized that current self-reported human health outcomes would be related to pet illness perceived to be caused by the FWC (April 2014–October 2015) cross-sectionally and longitudinally. One objective was to evaluate associations of human exposure to unfiltered tap water during the FWC with the perception the FWC caused an exposed pet to be ill (perceived pet health). Our second objective was to evaluate associations between perceived pet health and four self-reported outcomes (general health, physical health, mental health, and quality of life) among adults using both cross-sectional and longitudinal data from the Flint Registry.

## 2. Materials and Methods

The Flint Registry is a public health surveillance system that longitudinally follows individuals exposed to the FWC. The Flint Registry enrolled 13,973 adults whose identities were subsequently verified by probabilistic matching to at least one of three states of Michigan administrative datasets (Medicaid, Vital Records, and the Michigan Care Improvement Registry) from 1 December 2018, through 31 December 2022. Data included in this study were collected approximately 4–8 years after the date of the initial water switch and 2–6 years after the declaration of federal emergency. Participants enrolled by completing a baseline survey and those enrolled in the registry for at least 1 year were eligible to complete a follow-up survey 1 year later. The 1-year follow-up survey closed 31 July 2022, and was completed by 4892 adults. To be eligible for this study, participants had to live in the city of Flint during the water switch and have a pet in the household during the FWC. Additionally, they had to have complete data for pet’s perceived health variables, at least one of the human health-and-quality-of-life-related outcomes at baseline, and all covariates, which included age, race/ethnicity, annual household income, marital status, and use of tap water for drinking during the water switch.

### 2.1. Perceived Pet Health

Throughout its development and launch, the Flint Registry sought feedback from participants about topics that were important to them [17]. Community feedback indicated concern about the impact of the FWC on pets. The Flint Registry developed two questions administered in the baseline survey to evaluate pet ownership and pet health impacts that were used in this study: the first question asked, “Between April 2014 and October 2015, did your household have a pet?” (yes or no), and the second question asked, “Do you believe contact with Flint water, any time between April 2014 through October 2015, caused your pet to be sick?” (yes or no). Perceived pet health was categorized as “belief that pets in the house were made ill by water exposure” and “no belief that pets in the house were made ill”. Diagnostic confirmation of illness was not available.

### 2.2. Human Consumption of Unfiltered Tap Water

The Flint Registry questionnaire asked participants about the frequency of use of unfiltered tap water during the FWC for drinking, cooking, showering, washing dishes, and brushing teeth. The types and frequency of use were highly correlated, and so, for this analysis, the frequency of unfiltered tap water for drinking was used to estimate human exposure to unfiltered tap water. Frequency was reported as daily, less than daily, and never. We assumed drinking water source for respondents reflected their pets’ water source during the FWC.

### 2.3. Outcomes

Our outcomes were self-reported measures adapted from the PROMIS Global Health Scale v1.2 [18]. We asked participants at baseline, and at 1-year follow-up, how they rated, in general, their overall health, physical health, mental health, and quality of life on a five-point scale (poor, fair, good, very good, and excellent). We dichotomized the outcome measures into poor/fair and good or better.

### 2.4. Analysis

We used means (standard deviations) and frequencies (percentages) to describe the total population and to stratify the four outcome measures at baseline and at follow-up by perceived pet health. We used adjusted logistic regression models to estimate odds ratios and 95% confidence intervals (95% CI) for the associations of pet owner exposure to unfiltered tap water during the water switch and perceived pet health, and perceived pet health with outcome measures at baseline, while adjusting for covariates (age, race/ethnicity, annual household income, marital status, and use of tap water for drinking during the water switch). We also estimated associations of baseline perceived pet health with human health outcomes 1 year later to address the lack of temporality in the cross-sectional analyses of baseline data. We allowed denominators to vary for each outcome. Analyses were performed in SAS (SAS 9.4, SAS Institute, Cary, NC, USA). As a sensitivity analysis, we generated models that included participants that had both baseline and follow-up data to evaluate associations of baseline pet health with the four health-related outcomes (i.e., they included pet owners who had 1-year follow-up data on health outcomes so that we could compare models across time).

## 3. Results

As of 31 December 2022, 12,509 participants had completed the baseline survey and been validated through external data sources. Of these, 7130 did not have a pet living in the household during the FWC, 1657 were missing data about pet health, and 458 were missing data for covariates. Our baseline sample included 3264 adults with complete data for perceived pet health and covariates, and the 1-year follow-up sample included 1172 adults. Pet owners’ mean age was 47.3 years (standard deviation: 15.3) (Table 1), and 48.3% (*n* = 1575) reported their race as white, 39.2% (*n* = 1280) reported their race as Black, 68.5% reported their gender as female (*n* = 2236), and 35.6% reported a household income of less than USD 12,000 per year (*n* = 1163). The majority of pet owners lived in Flint for the entire duration of the water switch (full 18 months: *n* = 2985, 91.5%) and reported that they drank unfiltered tap water every day during the water switch (*n* = 2744, 84.1%).

### 3.1. Unfiltered Tap Water Consumption by Humans and Perceived Pet Health

At baseline, more than 60% of pet owners reported that they believed exposure to contaminated water made their pet ill (*n* = 2099, 64.3%) (Table 2). For pet owners who drank unfiltered tap water daily during the water switch, 67.2% (*n* = 1843) reported they believed exposure to Flint water made their pet ill. In comparison, 38.2% (*n* = 79) of pet owners who reported never drinking unfiltered tap water during the switch believed their pet was made ill by the water. Adjusted models showed Flint Registry pet owners who reported drinking the tap water every day were 3.12 (95% CI: 2.33–4.23) times as likely to report that their pet had been made ill by the FWC compared to pet owners who reported never drinking the water, and pet owners who reported drinking the water less frequently than every day had 2.05 (95% CI: 1.42–3.00) higher odds of reporting they believed the FWC made their pet ill (Table 2).

### 3.2. Perceived Pet Health and Human Health Outcomes at Baseline

**Figure 1:**
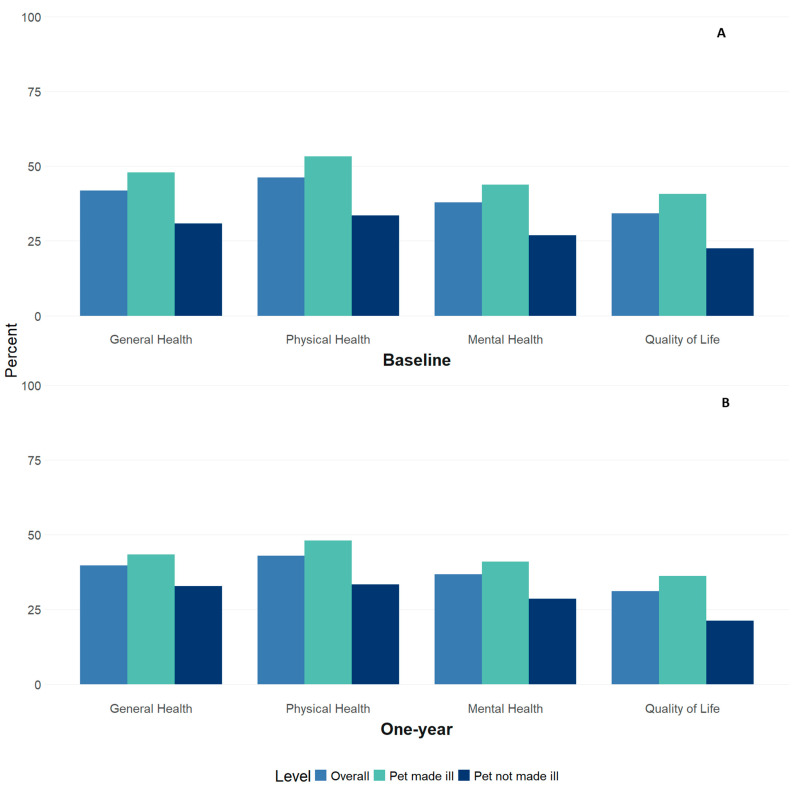
Panel (**A**) shows the percentages of adult Flint Registry pet owners who reported poor or fair health overall (light blue bar), among those who reported they believed exposure to Flint water made their pet ill (light green bar), and among those who did not believe exposure to Flint water made their pet sick (dark blue bar) for self-reported perceptions of general health (*n* = 3238), physical health (*n* = 3216), mental health (*n* = 3220), and quality of life (*n* = 3231) at baseline. Panel (**B**) shows the percentages of adult Flint Registry pet owners who reported poor or fair health overall (light blue bar), among those who reported they believed exposure to Flint water made their pet ill (light green bar), and among those who did not believe exposure to Flint water made their pet sick (dark blue bar) for self-reported perceptions of general health (*n* = 1171), physical health (*n* = 1165), mental health (*n* = 1166), and quality of life (*n* = 1169) at 1-year follow-up.

At baseline, 41.9% (*n* = 1355) of pet owners reported their general health as poor or fair, 46.2% (*n* = 1486) reported poor or fair physical health, 37.9% (*n* = 1219) reported their mental health as poor or fair, and 34.2% (*n* = 1106) reported poor or fair quality of life (Figure 1A). Adjusted odds ratios demonstrated that pet owners who reported their pet was made ill by the Flint water had about a two-fold increase in odds of reporting poor or fair health or quality of life, regardless of outcome (Table 3). The effect sizes were largest for physical health (odds ratio: 2.15, 95% CI: 1.83–2.52) and for quality of life (odds ratio: 2.16, 95% CI: 1.82–2.57). Effect sizes were similar when the sample was limited to pet owners who also had 1-year follow-up data.

### 3.3. Perceived Pet Health and Human Health Outcomes at Follow-Up

At 1-year follow-up, 39.7% (*n* = 465) of pet owners reported poor or fair general health, 43.0% (*n* = 501) reported poor or fair physical health, 36.7% (*n* = 428) reported poor or fair mental health, and 31.1% (*n* = 363) reported their quality of life as poor or fair (Figure 1B). One-year follow-up odds ratios were attenuated, though still statistically significant, and indicated that pet owners who believed contaminated Flint water had made their pets ill had a 1.5-to-2-fold higher odds of poor or fair health and quality of life outcomes compared to those who did not believe the Flint water had made their pets ill (Table 3). Effect sizes for physical health (odds ratio: 1.60, 95% CI: 1.22–2.10) and quality of life (odds ratio: 1.92, 95% CI: 1.42–2.60) remained the strongest associations.

## 4. Discussion

In our study, human water consumption was associated with perceived pet health during the FWC. In turn, the belief that pets were made sick by exposure to contaminated Flint water was associated with self-reported poor and fair human health and quality of life measured 4–8 years after the initial Flint water switch in both cross-sectional and longitudinal analysis. These findings support the concept of pets as early and vulnerable victims of environmental exposures and point to their potential role as sentinels of subsequent human health problems.

We found that perceived harm to pets from contaminated Flint water exposure was associated with poor or fair measures of health and quality of life among Flint Registry participants who had household pets during the water switch. While we cannot identify how pet owners’ post-disaster health trajectories were affected by the health trajectories of their pets, our results suggest that both physical and emotional influences on health may be at work. The strength of the association for the general physical health outcomes suggests that pets’ perceived health could act as a sentinel event for later human health, while the quality of life and mental health effects point to sustained emotional impacts of the FWC. Further research is necessary to confirm these hypotheses.

Pets are believed to influence human health through physical and emotional pathways [4]. During disasters, pet owners make safety decisions about their pets with implications for their own physical health [4,15]. Pet death related to disasters is associated with post-disaster distress and suggests the emotional toll of disasters on pet owners may have long-term consequences [19]. However, pets may also provide some benefit during long-term recovery from disaster, such as findings from the Great East Japan Earthquakes of 2011 suggest [20], which showed pet owners exhibited more post-traumatic stress disorder symptoms immediately after the earthquakes than non-pet owners but fewer post-traumatic stress disorder symptoms approximately 4 years later [21]. Pet–human relationships were found to be highly valued during times of crisis and the human–pet bond supported recovery [7]. In the case of the FWC, pets may have been unknowingly exposed to contaminated water by their owners [14], as acknowledgement of water contamination did not occur until September 2015 [11], potentially resulting in feelings of guilt and emotional distress. However, pet owners may have changed water sources prior to the announcement of lead contamination. For example, residents had concerns about their water quality early in the water crisis, and boil water advisories occurred before lead contamination was made public [21,22]. Dogs tested for lead after the emergency declaration were found to have blood lead concentrations that decreased over time in part because pet owners started filtering tap water and switching from tap water to bottled water for their dogs [15]. In this case, it is possible that pet owners’ change in water source reduced their own exposure along with their pets’ exposure, which may have reduced physical harm and prevented feelings of guilt for unknowingly exposing pets. In this study, most of the Flint Registry pet owners reported drinking unfiltered tap water daily during the water switch. Those who reported drinking water less than daily had elevated odds of believing their pet was made sick by the contaminated Flint water, but the odds were not as high as those who reported drinking water daily. It is possible that changing their source of drinking water influenced both pet and human health through reducing physical exposure to lead and other contaminants and by eliminating mental distress causing harm, though we cannot test this hypothesis. Our results suggest that monitoring exposure status and health of pets during future water disasters may help prevent and/or identify human outcomes.

## 5. Limitations

Although the sample size is large, this sample of Flint Registry pet owners only reflects the experience of those who reported they had a household pet during the Flint water switch. It is important to note the Flint Registry sample is a convenience sample and may not be representative of the entire target population of those exposed. We could not evaluate how pet ownership varied by pet type because we asked broadly about household pets but did not collect data on the type of pets. We also lacked data on human and pet lead exposure, pet exposure to unfiltered tap water, and pet health diagnoses and outcomes beyond what is reported herein. As such, this analysis could not evaluate changes to pet health that were the direct result of exposure to contaminated water, and we could not assess the causal relationship of perceived pet health with emotional and/or physical human health and quality-of-life outcomes. There is potential that pet owners’ perceptions of their pets’ health were reflections of their own health at the time of reporting (e.g., reverse causation). We looked at associations in three groups: baseline survey comparisons between pet and pet owner health, comparisons between baseline pet health and pet owner health 1 year later, and baseline data in the sample who completed a baseline and 1-year survey. We noted that the effect sizes of baseline pet health and 1-year human health outcomes were attenuated from the baseline-only models. This may be the result of reverse causation and highlights the need for tracking pet health after disasters in order to better characterize the relationship between pet health and human health. Finally, we cannot discern pathways (physical or emotional) by which perceived pet health impacted human health outcomes after the FWC.

## 6. Conclusions

Environmental disasters like the FWC highlight the One Health approach that the health of animals and people are interconnected. Our study showed that adults who consumed unfiltered tap water during the FWC reported worse perceived pet health, which was then correlated longitudinally with poor adult health. This research underscores the role of public health registries in the identification of unique challenges of subgroups impacted by environmental disasters like the FWC and the importance of pet surveillance to identify emerging human health outcomes.

## Figures and Tables

**Table 1:** Descriptive baseline characteristics of Flint Registry participants who had a pet during the Flint water crisis.

Characteristic	Frequency	Percent
*n*	3264	
Mean Age (standard deviation)	47.3	15.3
Race and Ethnicity		
White	1575	48.3
Black	1280	39.2
Other *	67	2.1
Multiracial	181	5.6
Latinx	161	4.9
Gender		
Female	2236	68.5
Male	1020	31.3
Other	8	0.3
Household Income		
<USD 12,000 per year	1163	35.6
USD 12,000–<USD 25,000	882	27.0
USD 25,000–<USD 50,000	770	23.6
USD 50,000–<USD 75,000	253	7.8
USD 75,000 and higher	196	6.0
Time living in Flint during the water crisis		
Less than the full 18 months	279	8.6
The full 18 months	2985	91.5
Marital Status		
Married	953	29.2
Widowed	199	6.1
Divorced	613	18.8
Separated	147	4.5
Never married/single	1352	41.4
Drank unfiltered tap water during the water crisis		
Every day	2744	84.1
Less than every day	313	9.6
Never	207	6.3

* Other single race includes: Native American/Alaska Native, Asian, Native Hawaiian/Other Pacific Islander, Middle Eastern/North African.

**Table 2:** Frequency and adjusted odds ratios for pet owners’ baseline perceived pet health and unfiltered tap water exposure during the Flint water crisis.

	Pet Made Ill	Pet Not Made Ill	Adjusted Odds Ratio (95% CI)
Perceived pet health (*n* = 3264)	2099 (64.3)	1165 (35.7)	-
Frequency of drinking unfiltered tap water during the water switch			
Every day	1843 (67.2)	901 (32.8)	3.12 (2.33–4.23)
Less than every day	177 (56.6)	136 (43.4)	2.05 (1.42–3.00)
Never	79 (38.2)	128 (61.8)	Reference

Participants with missing water exposure data, pet data, or covariate data were excluded. Model was adjusted for age at baseline, race and ethnicity, gender, household income, time living in Flint during the water switch, and marital status. Abbreviation: CI, Confidence Interval.

**Table 3:** Odds ratios for cross-sectional and longitudinal estimates of association of perceived pet illness with self-reported health outcomes.

Self-Reported Health Outcomes	Denominator	Adjusted Odds Ratio (95% CI)
Baseline (*n* = 3264)		
General Health Fair or Poor	3238	1.92 (1.63–2.26)
Physical Health Fair or Poor	3216	2.15 (1.83–2.52)
Mental Health Fair or Poor	3220	1.97 (1.67–2.32)
Quality of Life Fair or Poor	3231	2.16 (1.82–2.57)
Baseline for 1-Year Follow-up Sample (*n* = 1172)		
General Health Fair or Poor	1171	1.83 (1.38–2.43)
Physical Health Fair or Poor	1165	2.12 (1.61–2.79)
Mental Health Fair or Poor	1166	1.87 (1.41–2.49)
Quality of Life Fair or Poor	1169	2.57 (1.89–3.50)
1-Year Follow-up (*n* = 1172)		
General Health Fair or Poor	1171	1.32 (1.00–1.74)
Physical Health Fair or Poor	1165	1.60 (1.22–2.10)
Mental Health Fair or Poor	1166	1.50 (1.14–1.98)
Quality of Life Fair or Poor	1169	1.92 (1.42–2.60)

Denominators were allowed to vary for each outcome. Participants with missing pet data or covariate data were excluded. Models were adjusted for age, race/ethnicity, annual household income, marital status, and use of tap water for drinking during the water switch. Abbreviations: CI, confidence interval.

## Data Availability

Data are available upon reasonable request. Requested data may be provided after IRB approval and appropriate data use agreements have been obtained.
